# Genetic Mapping and Characterization of the Clubroot Resistance Gene *BraPb8.3* in *Brassica rapa*

**DOI:** 10.3390/ijms251910462

**Published:** 2024-09-28

**Authors:** Liyan Kong, Yi Yang, Yufei Zhang, Zongxiang Zhan, Zhongyun Piao

**Affiliations:** 1 Molecular Biology of Vegetable Laboratory, College of Horticulture, Shenyang Agricultural University, Shenyang 110866, China; 2 Guangdong Key Laboratory for New Technology Research of Vegetables, Vegetable Research Institute, Guangdong Academy of Agricultural Sciences, Guangzhou 510640, China

**Keywords:** clubroot, *Plasmodiophora brassicae*, mapping, *Brassica rapa*

## Abstract

Clubroot, a significant soil-borne disease, severely impacts the productivity of cruciferous crops. The identification and development of clubroot resistance (CR) genes are crucial for mitigating this disease. This study investigated the genetic inheritance of clubroot resistance within an F_2_ progeny derived from the cross of a resistant parent, designated “377”, and a susceptible parent, designated “12A”. Notably, “377” exhibited robust resistance to the “KEL-23” strain of *Plasmodiophora brassicae*, the causative agent of clubroot. Genetic analyses suggested that the observed resistance is controlled by a single dominant gene. Through Bulked Segregant Analysis sequencing (BSA-seq) and preliminary gene mapping, we localized the CR gene locus, designated as *BraPb8.3*, to a 1.30 Mb genomic segment on chromosome A08, flanked by the markers “333” and “sau332-1”. Further fine mapping precisely narrowed down the position of *BraPb8.3* to a 173.8 kb region between the markers “srt8-65” and “srt8-25”, where we identified 22 genes, including *Bra020861* with a TIR-NBS-LRR domain and *Bra020876* with an LRR domain. Quantitative reverse transcription polymerase chain reaction (qRT-PCR) analyses confirmed that both *Bra020861* and *Bra020876* exhibit increased expression levels in the resistant parent “377” following inoculation with *P. brassicae*, thereby underscoring their potential as key genes implicated in *BraPb8.3*-mediated clubroot resistance. This study not only identifies molecular markers associated with *BraPb8.3* but also enriches the genetic resources available for breeding programs aimed at enhancing resistance to clubroot.

## 1. Introduction

Chinese cabbage (*Brassica rapa* subsp. *pekinensis*) is a vital vegetable crop within the Brassicaceae family that holds significant economic and nutritional importance in various regions across the globe [1]. This species is widely cultivated due to its rapid growth cycle and its strong adaptability to various environmental conditions. One of the primary challenges faced during Chinese cabbage cultivation is its susceptibility to various diseases, with clubroot caused by the obligate pathogen *Plasmodiophora brassicae* being one of the most devastating [2,3]. Clubroot disease is characterized by the enlargement of plant roots, which impedes the absorption of water and nutrients and, in severe cases, can result in plant death [4]. The life cycle of *P. brassicae* is not well understood due to its obligate intracellular biotrophic nature [5]. Current management strategies for clubroot include field practices, chemical interventions, biological measures, and the development of resistant cultivars [6]. Among these, the identification of resistance genes from naturally immune plant materials and the breeding of clubroot-resistant cultivars are the most efficient and sustainable approaches to prevent the spread of the disease [7]. Traditional breeding methods have been employed to introduce resistance traits, but these can be time-consuming and labor-intensive. The advent of molecular biology and genomics has opened new avenues for the identification of genetic markers associated with disease resistance, facilitating more efficient and targeted breeding strategies [8].

Plants have evolved an immune system to defend against pathogens, comprising two primary branches: pattern-triggered immunity (PTI) and effector-triggered immunity (ETI) [9]. PTI is initiated by cell surface-localized pattern-recognition receptors (PRRs) that detect conserved microbial patterns. In contrast, ETI is activated in response to pathogen-derived effectors. Plants can develop resistance genes that recognize these effectors, triggering a robust immune response [10]. Most resistance genes encode proteins of the TIR-NBS-LRR (Toll-interleukin-1 receptor-like domain-nucleotide binding site-leucine-rich repeat) family [11]. *In B. rapa*, numerous CR genes have been identified, such as *Crr2* [12], *PbBa1.1* [13], and *QS_B1.1* [14] on chromosome A01; *Crc* [15] and *Rcr8* [16] on chromosome A02; *Cra* [17], *Crb* [18], *Crd* [19], *Crk* [15], *Crr3* [20], *PbBa3.1*, *PbBa3.2*, *PbBa3.3* [13], *Rcr1* [21], *Rcr2* [22], *Rcr4* [16], *Rcr10^ECD0^*^1^ [23], *CRA3.7* [7] on chromosome A03; *CrrA5* [24] on chromosome A05; *Crr4* [25] on chromosome A06; *qBrCR38-1* on chromosome A07 [26]; and *Crr1a* and *Crr1b* [27], *CRs* [28], *PbBa8.1* [13], *qBrCR38-2* [26], *Rcr3,* and *Rcr9^wa^* [29] on chromosome A08. Notably, the majority of CR loci are concentrated on chromosomes A03 and A08, with no identified loci on chromosomes A04, A09, and A10. Among these, only *CRa* and *Crr1a* have been isolated and functionally validated. These CR genes confer distinct resistance to various pathotypes of *P. brassicae.* For example, *CRa* from “T136-8” is resistant to the M85 isolate (race 2) of *P. brassicae* [17]; *CRb* from “CR Shinki” is resistant to races 2, 4, and 8 [18]; *CRd* from “85-74” is resistant to LAB-19 isolate (race 4) [19]; and *Crr1a* from “G004” confers resistance to the Ano-01 isolate [27]. Thus, identifying new CR genes or alleles with resistance to different pathotypes of *P. brassicae* is essential for managing clubroot disease in Chinese cabbage and addressing the pathogen’s rapid mutation, which can lead to resistance breakdown.

Molecular marker selection (MAS) is a powerful tool in plant breeding that uses DNA markers to screen for agriculturally important traits, enhancing the efficiency and effectiveness of trait selection [30]. Common molecular markers include simple sequence repeats (SSRs), inter simple sequence repeats (ISSRs), insertions and deletions (InDels), and single-nucleotide polymorphisms (SNPs) [31,32,33]. MAS has been widely applied in the improvement and breeding of various crops [34,35,36,37]. Identifying molecular markers linked to CR genes or loci can significantly speed up the breeding process of Brassica crops [38]. By combining multiple CR genes within a single cultivar, broad-spectrum resistance to clubroot can be achieved [39]. Currently, MAS is extensively used for transferring CR genes in Brassicaceae crops [38,40,41].

To identify novel clubroot resistance (CR) genes in Chinese cabbage, this study established a new segregating population from the sensitive cultivars “12A” and “377” to elucidate the genetic basis of *BraPb8.3* resistance. By employing Bulked Segregant Analysis sequencing (BSA-seq) on two extreme pools, in conjunction with genetic mapping, we identified the *BraPb8.3* resistance locus. The outcomes of this research provide a robust tool for the breeding of clubroot-resistant Chinese cabbage varieties.

## 2. Results

### 2.1. Phenotype Evaluation and Genetic Analysis

**Figure 1:**
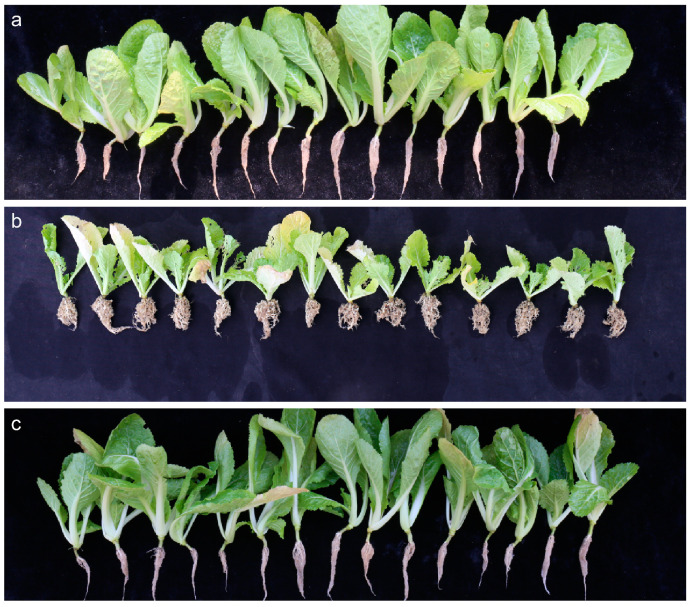
Phenotype of two parental Chinese cabbage lines and the F_1_ offspring after inoculation with “KEL-23” isolate. (**a**) Resistance parent “377” (P1). (**b**) Susceptible parent “12A” (P2). (**c**) The F_1_ offspring individuals.

To delineate the genetic inheritance of the clubroot disease-resistance locus *BraPb8.3*, a cross was performed between the resistant *B. rapa* cultivar ‘377’ (Figure 1a) and the susceptible cultivar ‘12A’ (Figure 1b), yielding F_1_ and F_2_ progenies. Inoculation tests with *P. brassicae* on 28 F_1_ plants uniformly demonstrated resistance, with no susceptible individuals observed (Figure 1c). Within the F_2_ population of 470 plants, 357 showed resistance and 113 showed susceptibility, resulting in a segregation ratio of 3:1, as detailed in Table 1. The chi-square test was applied to assess the fit to Mendelian inheritance patterns, yielding a calculated χ^2^ value of 0.18, which is substantially lower than the critical value of 3.84 at the *p* = 0.05 level (χ^2^_0.05_ = 3.84). These findings strongly suggest that the resistance to clubroot disease in the *B. rapa* cultivar ‘377’ is governed by a single dominant gene.

### 2.2. BSA-Seq Data Analysis

BSA-seq was utilized to identify the genomic region associated with clubroot resistance, with the sequence raw data deposited under the SRA accession SRX24034031 and SRX24034032. This approach involved sequencing two DNA pools: the resistant pool (R-pool) and the susceptible pool (S-pool), as detailed in Table 2. The R-pool yielded 72,065,694 clean reads, while the S-pool produced 67,798,184 clean reads, corresponding to 10,553,867,052 and 9,861,094,696 clean bases, respectively. Of these, 10,004,832,128 bases from the R-pool and 9,374,230,064 bases from the S-pool were successfully mapped to the reference genome, achieving mapping rates of 94.80% and 95.06%, respectively. The duplication rates were 17.41% for the R-pool and 17.60% for the S-pool. The high-quality base percentages were consistent at 67.00% for the R-pool and 67.46% for the S-pool. The average depth of coverage on the reference genome was 28.45 for the R-pool and 26.65 for the S-pool. Furthermore, the coverage rates, which represent the proportion of the reference genome covered at least four times, were 94.88% for the R-pool and 94.97% for the S-pool. These metrics indicate a high degree of consistency between the sequencing data of the R-pool and S-pool, providing a reliable foundation for identifying candidate genomic regions linked to clubroot disease resistance.

**Figure 2:**
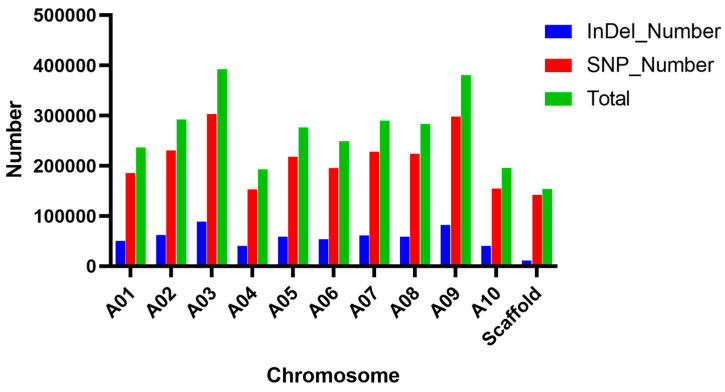
Distribution of SNPs and InDels.

Analysis of SNPs and InDels across various chromosomes revealed significant genomic variation within the studied population, as illustrated in Figure 2. The total count of identified variants, including both SNPs and InDels, across all chromosomes reached 2,941,021, which is indicative of considerable genomic diversity. Notably, chromosome A03 exhibited the highest variant count, suggesting regions of enhanced genetic diversity or increased mutational activity relative to other chromosomes. Additionally, the prevalence of SNPs over InDels across most chromosomes indicated that single nucleotide changes are more common than insertion–deletion events in this population. Furthermore, there was observed variability in the count of variants among different chromosomes; for instance, chromosomes A03 and A09 showed significantly higher variant counts than others. This variability may be attributed to differences in recombination rates, selective pressures, or other genomic factors that influence mutation rates and genetic diversity.

### 2.3. Euclidean Distance Analysis and Prediction of Candidate Areas

**Figure 3:**
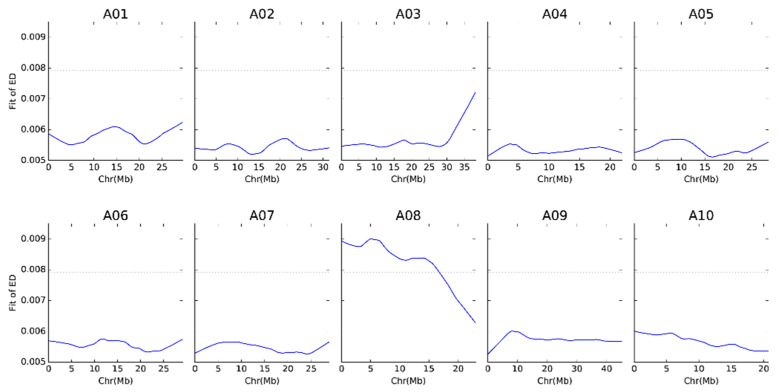
The Euclidean distance calculations of SNPs between the R- and S-pool samples. The horizontal axis indicates the physical distances across each chromosome, and the vertical axis represents the fit values of the ED values raised to the fourth power. The solid blue line corresponds to the actual fitting curve of the SNPs, while the dashed black line denotes the threshold line.

The Euclidean distance (ED) analysis was conducted to assess the genetic distance between two samples based on SNP difference. Out of the 2,941,021 variants identified, a subset comprising 942,954 SNPs was selected for the calculation of the SNP index. As a result, a candidate genomic region was identified on chromosome A08, spanning from position 788 to 16,743,576, with a threshold cut-off value of 0.0079 for the ED analysis (Figure 3).

### 2.4. Preliminary Mapping of BraPb8.3

**Figure 4:**
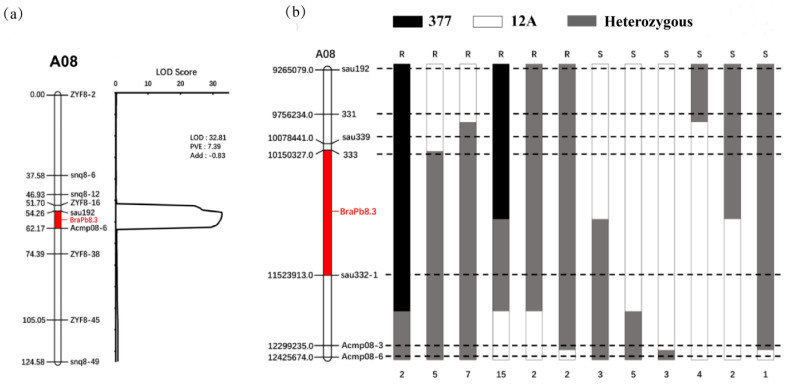
Preliminary mapping of the *BraPb8.3* gene locus. (**a**) Genetic mapping and Logarithm of Odds (LODs) threshold analysis for *BraPb8.3* gene locus. (**b**) Preliminary mapping of the *BraPb8.3* gene, including analysis of recombinant genotypes. Marker genotypes ‘377’ and ‘12A’ are represented in black and white, respectively, with heterozygous genotypes depicted in gray. Phenotypic classifications are indicated by ‘R’ for resistance and ‘S’ for susceptibility. The Arabic numerals beneath the figure indicate the count of recombinants.

To accurately pinpoint the chromosomal location of the *BraPb8.3* gene locus, a cohort of 470 F_2_ individual plants was utilized for preliminary mapping. Initially, 120 plants were randomly selected to form the mapping population. Linkage analysis, conducted using nine marker pairs in the JoinMap software, demonstrated a significant association with the target trait. The marker sequences are detailed in Table S3. Subsequent analysis, incorporating post-inoculation phenotypic evaluation of *P. brassicae* and quantitative trait loci (QTL) analysis with the IciMapping software, identified a significant SNP site characterized by a LODs score of 32.81, a contribution rate of 7.39%, and an additive effect of −0.83. This SNP site was located between the markers “sau192” and “Acmp08-6”, thereby mapping the *BraPb8.3* gene locus to chromosome A08 (Figure 4a).

To refine the localization interval of the *BraPb8.3* gene locus, the remaining 350 F_2_ individuals were subjected to a recombinant screening process using the flanking markers “sau192” and “Acmp08-6”. This screening identified 51 recombinant individuals. Subsequent genotyping of these individuals with a refined set of markers, including “331”, “sau339”, “333”, “sau332-1”, and “Acmp08-3”(Table S3), along with phenotypic analysis, allowed for the preliminary mapping of the *BraPb8.3* locus to a 1.30 Mb interval between the markers “333” and “sau332-1” (Figure 4b).

### 2.5. Fine Mapping of BraPb8.3

**Figure 5:**
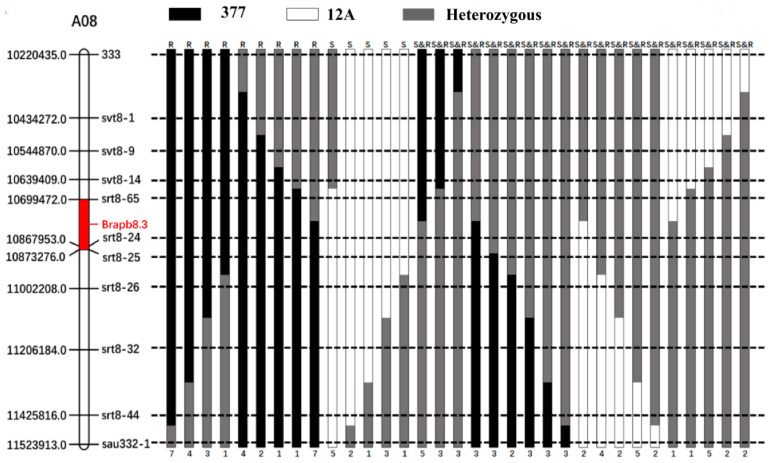
Genotypic and phenotypic analysis of 96 recombinants from the fine mapping study. Genotypes for markers ‘377’ and ‘12A’ are depicted in black and white, respectively, with heterozygous genotypes indicated in gray. Phenotypic classifications are denoted by ‘R’ for resistance, ‘S’ for susceptibility, and ‘S&R’ for families displaying mixed phenotypes of both resistance and susceptibility. The Arabic numerals beneath the figure indicate the count of recombinants.

To further refine the localization interval of the *BraPb8.3* gene locus, fine mapping was conducted. A cohort of 3000 F_2_ progenies was screened using the molecular markers “333” and “sau332-1”, which are linked to *BraPb8.3*, resulting in the identification of 96 recombinants. These recombinants were self-pollinated to generate F_3_ lineages, which were then inoculated with *P. brassicae* to assess resistance phenotypes. The phenotypic analysis revealed 30 families with pronounced resistance, 12 families with complete susceptibility, and 54 families showing intermediate levels of resistance. Subsequently, the 96 recombinants were genotyped using nine sets of polymorphic markers within the interval defined by “333” and “sau332-1” (Figure 5). By integrating phenotypic data from clubroot disease assessments with genotypic information, the *BraPb8.3* resistance locus was precisely mapped to a 173.8 kb segment flanked by the molecular markers “srt8-65” and “srt8-25” on chromosome A08.

### 2.6. Candidate Genes Analysis

Upon comparison with the Chiifu-401-42 reference genome, it was determined that the 173.8 kb region containing the *BraPb8.3* locus encompasses 22 genes (Table 3). Among these, the gene *Bra020861* is particularly noteworthy, as it encodes a disease-resistance protein of the TIR-NBS-LRR class, which implicates its potential role in conferring resistance to clubroot disease. Additionally, the *Bra020876* gene, which contains a leucine-rich repeat (LRR) domain, may also contribute to the resistance response against clubroot. Furthermore, several other genes within this region are known to be involved in stress responses, chromatin remodeling, DNA binding, and protein chaperoning. Notably, *Bra020860* encodes an F-box protein, which could be implicated in the protein degradation pathways associated with disease resistance. The presence of genes associated with calcium permeability, methionine sulfoxide reduction, and RNA polymerase subunits further indicated the involvement of diverse molecular pathways in the resistance to clubroot disease. Further functional characterization of these genes could elucidate the molecular underpinnings of clubroot resistance in Chinese cabbage. In this study, *Bra020861* and *Bra020876* were considered as candidate genes retained for further investigation.

Following a comparative sequence analysis of the *Bra020876* gene from the resistant parent “377” and the susceptible parent “12A,” seven nucleotide differences were identified. These variations included four transitions between cytosine (C) and thymine (T), two transitions between guanine (G) and adenine (A), and a single transversion from guanine (G) to thymine (T) (Supplementary Figure S1a). Moreover, the analysis revealed significant deletions in the *Bra020861* gene sequence of the resistant parent “377” compared to that of the susceptible parent “12A” (Supplementary Figure S1b).

### 2.7. Expression Analysis of Candidate Genes

**Figure 6:**
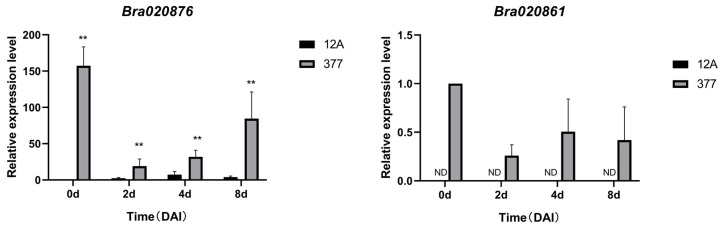
Relative expression levels of *Bra020861* and *Bra020876* genes in the roots of Chinese cabbage following inoculation with *P. brassicae.* The horizontal axis represents days after inoculation (DAI), with “ND” signifying that the gene expression was below the detection threshold. ** *p* < 0.01.

To elucidate the contribution of two candidate genes to clubroot resistance, their expression levels were quantified using quantitative reverse transcription PCR (qRT-PCR) (Figure 6). The qRT-PCR analysis revealed significantly higher expression of the *Bra020876* gene in the resistant line “377” compared to the susceptible line “12A”. Additionally, expression of the *Bra020861* gene was exclusively detected in the resistant line ‘377’. These findings collectively suggest that both *Bra020876* and *Bra020861* are likely to play a contributory role in the mediation of resistance against clubroot disease.

## 3. Discussion

Clubroot disease, caused by *P. brassicae*, poses a significant threat to cruciferous crops, impairing their ability to absorb water and nutrients [4]. The obligate biotrophic nature and intricate life cycle of *P. brassicae* present numerous challenges for the prevention and management of clubroot [5]. The exploration of clubroot resistance (CR) genes and the breeding for disease resistance are considered the most effective strategies for clubroot disease prevention [7,42,43]. Some resistant germplasms exhibit specificity to different pathotypes of *P. brassicae* [13,19,42]. Considering the frequent presence of multiple pathotypes of *P. brassicae* in the field, a germplasm with resistance to several pathotypes can more effectively prevent clubroot disease. The Chinese cabbage disease-resistant parent “377” utilized in this study exhibited robust resistance to pathotypes Pb2, Pb4, and Pb10 of *P. brassicae*, as defined by the sinitic clubroot differential set [44]. Therefore, identifying the resistant locus in “377” is of significant importance for breeding efforts against clubroot disease.

Numerous studies have shown that resistance within the A genome is commonly regulated by a major dominant locus [18,19,45]. In our study, the F_1_ hybrid derived from the resistant parent “377” and the susceptible parent “12A” exhibited clubroot resistance, with a segregation ratio of 3:1 for resistance and susceptibility observed in the F_2_ generation. This segregation pattern strongly suggests that clubroot resistance is controlled by a single dominant gene. BSA-seq is an efficient method for finely mapping the quantitative trait loci interval of target genes through the sequencing of mixed-DNA gene pools. This approach is widely employed in gene mapping studies [46,47,48,49,50]. In our research, we utilized the combination of BSA-seq with the ED algorithm to establish a threshold of 0.0079 for identifying candidate regions, which allowed us to pinpoint the clubroot-resistance gene to chromosome A08. Several studies have documented the presence of seven clubroot resistance (CR) loci on chromosome A08, spanning the 10.39–13.67 Mb region, including *CRs*, *PbBa8.1*, *Rcr3*, *Rcr9*, *Crr1*, *Rcr9^w^*^a^, and *CRA8.1* [13,28,29,51,52]. In this work, *BraPb8.3* was identified within the 10.69–10.87 Mb region of chromosome A08. Employing traditional preliminary and fine mapping approaches, we further refined the *BraPb8.3* locus to a 173.8 kb interval flanked by the “srt8-65” and “srt8-25” markers on chromosome A08. The development of molecular markers closely associated with target genes, coupled with molecular marker-assisted breeding techniques, provides powerful tools for combating clubroot disease [41,53]. The molecular markers ‘333’ and ‘sau332-1’ have demonstrated exceptional efficacy in selecting clubroot-resistant individuals within the F_2_ population, indicating their close linkage to the clubroot resistance (CR) loci. Furthermore, the molecular markers ‘srt8-65’ and ‘srt8-25’ have been identified as closely linked to the *BraPb8.3* locus, suggesting their potential utility in assisting breeding programs focused on enhancing clubroot resistance in Chinese cabbage

The majority of characterized disease resistance (R) genes are known to feature the TIR-NBS-LRR domains [11,41]. In our study, within the finely mapped region of the Chiifu-401-42 genome, *Bra020861* was identified as a TIR-NBS-LRR type R gene, while *Bra020876* was found to possess an LRR domain, highlighting their potential roles in disease resistance mechanisms. Sequence analysis revealed variations between these candidate genes in the resistant and susceptible parents. Additionally, relative gene expression analysis showed that *Bra020861* was highly expressed in the disease-resistant parent “377”, in contrast to being either undetectable or expressed at very low levels in the susceptible parent “12A”. On the other hand, *Bra020876* was upregulated in the resistant parent “377”. Together, these results suggest that *Bra020861* and *Bra020876* are likely key contributors to clubroot disease resistance.

In summary, the clubroot resistance locus *BraPb8.3* has been precisely located within a 173.8 kb interval between markers “srt8-65” and “srt8-25” on chromosome A08 of *B. rapa*. Within this delineated region, two genes featuring the TIR-NBS-LRR or LRR domains were identified. Through sequence comparison and expression analysis, *Bra020861* and *Bra020876* have emerged as significant candidate genes for the *BraPb8.3*-mediated resistance to clubroot disease. These findings necessitate further investigation to confirm the roles of these genes in disease resistance. Importantly, our research not only identified a key clubroot resistance locus in *B. rapa* but also established molecular markers linked to novel CR genes. This advancement significantly contributes to our understanding of the genetics and management strategies against clubroot disease. Furthermore, our work provides valuable genetic resources that can be utilized for the control of clubroot and offers potent molecular markers to enhance breeding programs in Chinese cabbage.

## 4. Materials and Methods

### 4.1. Plant Materials and P. brassicae Inoculation

The Chinese cabbage-resistant inbred line ‘377’ was hybridized with the susceptible inbred line ‘12A’ to generate a segregating F_1_ generation. This F_1_ generation was subsequently self-pollinated to yield an F_2_ generation consisting of 3470 individuals. In order to pinpoint the clubroot resistance (CR) locus, the F_1_, F_2_, and F_3_ populations were inoculated with the *P. brassicae* isolate “KEL-23” (Pb4). For inoculation, *P. brassicae* isolates were extracted from homogenized clubbed Chinese cabbage roots and then diluted to a concentration of 1 × 10^7^ spores/mL. Following this, 1 mL of the suspension was introduced into the soil near the roots of each 2-week-old seedling. The assessment of clubroot disease resistance was conducted at five weeks post-inoculation.

### 4.2. BSA-Seq Analysis

DNA from “377”, “12A”, and the 3470 F_2_ progenies was extracted using a modified CTAB method [54]. Two DNA pools from the F_2_ generation, one of susceptible (S-pool) and one of resistant individuals (R-pool), each containing DNA from 20 individuals, were sequenced on an Illumina HiSeq 2500 (Annogene, Beijing, China). After cleaning and aligning the sequences to the *B. rapa* reference genome (http://brassicadb.cn/#/ (accessed on 23 September 2024)), we conducted a variant analysis with GATK [55], filtering for quality and depth. ANNOVAR was used for annotation [56]. SNPs and InDels were further screened, with a focus on significant variations, leveraging Euclidean distance (ED) for differential analysis [57], and we set a threshold based on the standard deviation.

### 4.3. Molecular Marker Development

This study utilized Bulk Segregant Analysis sequencing (BSA-seq) and employed the Chiifu-401-42 reference genome sequence, sourced from the Brassica Database (BRAD, http://brassicadb.cn/#/ (accessed on 23 September 2024)), for the purpose of developing molecular markers within the *BraPb8.3* candidate region. SSR and InDel markers were designed to facilitate quantitative trait loci (QTL) mapping and fine mapping. A subset of the SSR markers was generated using SSR Hunter version 1.3 software [58], while other SSR markers have been previously established by our research team [13,59]. The development of InDel markers adhered to the methodologies outlined in recent research [7].

### 4.4. Construction of Genetic Linkage Map and QTL Analysis

The genetic linkage map construction was performed using the JoinMap version 4.0 software [60]. This involved integrating genotype data from the F_2_ population with the corresponding markers. Recombination frequencies were calculated using the Kosambi mapping function. A threshold LODs score of 3.0 was applied to guide the generation of the genetic linkage map. Simultaneously, the QTL IciMapping software version 4.0 was utilized to analyze phenotypic responses in individual F_2_ progenies post *P. brassicae* inoculation. This analysis included computing LODs scores and identifying QTL linked to disease resistance.

### 4.5. Fine Mapping of BraPb8.3

Two molecular markers, “333” and “sau332-1”, which are closely linked to the *BraPb8.3* locus, were employed to select recombinant individuals. Following this selection process, 96 recombinant individuals were identified from an F_2_ population of 3000 individuals, and these individuals were subsequently self-pollinated to generate F_3_ families. Both parental lines and the 96 F_3_ family lines underwent inoculation with the *P. brassicae* isolate “KEL-23”. By integrating the clubroot resistance phenotype observed in the F_3_ families with the genotype of the recombinant individuals, the clubroot resistance gene *BraPb8.3* was finely mapped.

### 4.6. Prediction and Analysis of Candidate Genes

Candidate genes within the *BraPb8.3* fine mapping interval were predicted using the BRAD website. Subsequently, these genes were analyzed on the Pfam website (http://pfam.xfam.org/ (accessed on 23 September 2024)) to ascertain the presence of the TIR-NBS-LRR domain. Primers, outlined in Table S1 and designed based on the Chiifu-401-42 reference genome sequence, were used to amplify candidate genes from the genomic DNA of the disease-resistant parent ‘377’ and the susceptible parent ‘12A’. The PCR products were purified, ligated into the T vector (Takara, Dalian, China), then transformed into *Escherichia coli* strain DH5α, and selected for monoclonal colonies. These were subsequently subjected to Sanger sequencing to determine the nucleotide sequences of the cloned inserts. A comparative analysis of the sequencing data was performed to identify sequence variations potentially contributing to the observed phenotypic differences in disease resistance between the parental lines.

### 4.7. Total RNA Extraction and qRT-PCR

Total RNA was extracted from the roots of both parental lines using a TRIZOL reagent (Tiangen, Beijing, China), according to the manufacturer’s instructions. RNA isolation of high quality was then followed by the synthesis of first-strand complementary DNA (cDNA) using the FastKing RT Kit (Tiangen, Beijing, China). Quantitative Real-Time PCR (qRT-PCR) analysis was conducted using SuperReal PreMix Plus (Tiangen, Beijing, China). Primers for qRT-PCR were designed based on the reference genome sequence of Chiifu-401-42, with sequences provided in Table S2. The *18SrRNA* gene was chosen as the internal reference for normalization in Chinese cabbage. The relative expression levels of the genes of interest were determined using the 2^−ΔΔCT^ method [61].

## Figures and Tables

**Table 1:** Genetic analysis of clubroot resistance in the 377, 12A, F_1_, and F_2_ populations.

	Resistant Plant	Susceptible Plant	Theoretical Ratio	χ^2^	χ^2^_0.05_
377	36	0			
12A	0	36			
F1	28	0			
F2	357	113	3:1	0.18	3.84

**Table 2:** Quality control of sequencing data.

Sample	R-Pool	S-Pool
Genome Length	351,063,200	351,063,200
Clean Reads	72,065,694	67,798,184
Clean Bases	10,553,867,052	9,861,094,696
Mapped Bases	10,004,832,128	9,374,230,064
Mapping Rate (%)	94.80	95.06
Duplication Rate (%)	17.41	17.60
Uniq Rate (%)	67.00	67.46
Mean Depth	28.45	26.65
Coverage Rate (%) (>=4X)	94.88	94.97
Genome Length	351,063,200	351,063,200

**Table 3:** Characteristics of 22 predicted genes between molecular markers “srt8-65” and “srt8-25”.

Gene	Homologous Genes	Functional Annotations
*Bra020856*	AT4G22140	Encoding a chromatin remodeling factor that regulates flowering time
*Bra020857*	AT4G22120	Calcium-permeable stretch-activated cation channel
*Bra020858*	AT4G22100	Beta glucosidase 2
*Bra020859*	AT4G22080	Root hair specific 14
*Bra020860*	AT4G22060	F-box protein
*Bra020861*	AT3G25510	Disease resistance protein (TIR-NBS-LRR class) family protein
*Bra020862*	AT4G21910	MATE efflux family protein
*Bra020863*	AT4G21910	MATE efflux family protein
*Bra020864*	AT4G21895	DNA binding protein
*Bra020865*	AT4G21870	HSP20-like chaperone
*Bra020866*	AT4G21865	Hypothetical protein
*Bra020867*	AT4G32270	Unknown
*Bra020868*	AT4G21850	Methionine sulfoxide reductase B9
*Bra020869*	AT3G18550	Unknown
*Bra020870*	AT4G21810	DERLIN-2.1
*Bra020871*	AT4G21800	Conserved hypothetical ATP binding protein
*Bra020872*	AT4G21750	Encodes a homeobox protein similar to GL2
*Bra020873*	AT4G21710	Encodes the unique second-largest subunit of DNA-dependent RNA polymerase II
*Bra020874*	AT4G21710	Encodes the unique second-largest subunit of DNA-dependent RNA polymerase II
*Bra020875*	AT4G20930	Encodes a 3-hydroxyisobutyrate dehydrogenase
*Bra020876*	AT4G20940	Leucine-rich repeat N-terminal domain
*Bra020877*	AT4G20960	Cytidine and deoxycytidylate deaminase zinc-binding region

## Data Availability

The data supporting the results are included in this article. Additional relevant materials are available upon reasonable request from the corresponding author. The raw data from Bulked Segregant Analysis sequencing have been deposited at the NCBI Sequence Read Archive (SRA) repository under the accession numbers SRX24034031 and SRX24034032.

## Supplementary Materials

The following supporting information can be downloaded at https://www.mdpi.com/article/10.3390/ijms251910462/s1.
