# Supplementary material for: Genetic Mapping and Characterization of the Clubroot Resistance Gene BraPb8.3 in Brassica rapa
Source: Int J Mol Sci. 2024 Sep 28;25(19):10462. doi: 10.3390/ijms251910462 (PMC11477069; doi:10.3390/ijms251910462)
Supplement: Supplementary file 1 [file ijms-25-10462-s001.zip › ijms-3172209-supplementary.pdf]

Table S1 Primers sequence for amplifying candidate genes

Primer name	Primer	Sequences (5′-3′)
Bra020876	Forward	ATGGGGCAGCTTCCTTCACA
	Reverse	CTAAATTGAAGACAGGTCT
Bra020861-1	Forward	ATGGTGCCGAGATGTTCAAG
	Reverse	AGTAACCAGCAGGAGGCA
Bra020861-2	Forward	AAGCCTGACGACGTTGACGC
	Reverse	GCTATGTGAAGAAACAAAGCT
Bra020861-3	Forward	CTGCTTTTTCAACGGTGAAC
	Reverse	ACGAGATTAGTCATATTCC
Bra020861-4	Forward	GAAATTGGATCTCTCCAGAT
	Reverse	CTAAACCGGACTAAACCAGA

Table S2 Primers sequence for qRT-PCR of two candidate genes

Primer name	Primer	Sequences (5′-3′)
Bra020876-RT	Forward	TTCTCAGGCTCCCTTCCTAATA
	Reverse	CACTTATTGGCCCTGAGAGATT
Bra020861-RT	Forward	CTGAATCGAGAAGCAGTAGACC
	Reverse	CCGTGGCTCTGTAACTGAAATA

Table S3 InDel and SSR primers sequence information

Marker name	Primer	Sequences (5′-3′)
ZYF8-2	Forward	TCCGGTTTTTAATTGCGAGT
	Reverse	AGATTTGTAGGGGTTTTAAGAGTTC
snq8-6	Forward	CATTCGCTTCTCCACACAAA
	Reverse	AAAATGCCTCGTGTCGAGTT
snq8-12	Forward	ACGAAGGATCAAGGACGATG
	Reverse	TTTTGTAAGTTGTGCCGTCG
ZYF8-16	Forward	TCTGCACTCACTTGCTCCAC
	Reverse	CAGCACCCATCGATCACTTA
sau192	Forward	TCCCTCCTCTCTACGTCTTCTTC
	Reverse	CTTCTCTGTAACGGGCTTTGAC
Acmp08-6	Forward	CCATGACTAACTGAACCCTCGT
	Reverse	GGTGTGTGATTGTAGGGACCA
ZYF8-38	Forward	ACAGCTCCTCTTCCTCCTCC
	Reverse	GAAAGCTGTTCTCCACGAGC
ZYF8-45	Forward	CGACCCCCTTCATTAGTTGA
	Reverse	CTTCGTTTGCAACTTCCTCC
snq8-49	Forward	CGTGGTACATGAGCATCGTT
	Reverse	TTTTGAACCTTTGTTGTCTCCA
331	Forward	TTTTGGCCAAGATCGGTCGA
	Reverse	TCCTAGGCAAAAACCGGACC
sau339	Forward	CGGCGTTAAGGGTTTTAGCG
	Reverse	TGAGGATCGGTTTTCGGGTG
333	Forward	ACCTCATACGATGGTCTTCTGT
	Reverse	TTTTCTCTGGGGGCATGCTT
sau332-1	Forward	ACGCTGGTAGATATGTGGTCA
	Reverse	TCCCCCTTCAATGGAACCAC
Acmp08-3	Forward	GCCATGCCAGGTTTGCTTTT
	Reverse	AGTTCCAAAGCCGGATTCGT

(a)

Bra020876

```
12A     GAGTTATCCGGAGAGTTGCCTGGTTTTAATTATGTCTATGATCTTCAAGTGTTCAAGCTT
377     GAGTTATCCGGAGAGTTGCCTGGTTTTAATTATGTCTATGATCTTCAAGTGCTCAAGCTT
        *************************************************** ********

12A     AGCAACAACAGATTCTCAGGTTCACTTCCCAATAACTTGTTGAAAGGTGACTCTTTGCTT
377     AGCAACAACAGATTCTCAGGTTCACTTCCCAATAACTTGTTGAAAGGTGACTCTTTGCTT
        ************************************************************

12A     TTAGCGACGTTGGATTTAAGTGGCAACAATCTCTCAGGTATGCTCTTAGCTAGAGAGTGG
377     TTAGCGACGTTGGATTTAAGTGGCAACAATCTCTCAGGTATGCTCTTAGCTAGAGAGTGG
        ************************************************************

12A     GGATTGTTATATGCTAATGGTGGCACCAAGAAAACTAAAAATCTAACTCTTTTTGTTTGT
377     GGATTGTTATATGTTAATGGTGGCACCAAGAAAACTAAAAATCTAACTCTTTTTGTTTGT
        ************* **********************************************

12A     GTGGGAGAGCTGCAGTTTCTGGATGATTCTATCAAACTGACTCCAGAGGAGTTGTCGAGG
377     GTGGGAGAGCTGCAGTTTCTGGATGATTCTATCAAACTGACTCCAGAGGAGTTGTCTAGG
        ******************************************************** ***

12A     GCACCAGCTGAAGTCCTGGGAAGAAGTAGCCACGGGACTTCTTACAGAGCAACGCTTGAT
377     GCACCAGCTGAAGTCCTGGGAAGAAGTAGCCACGGGACTTCTTACAGAGCAACGCTTGAT
        ************************************************************

12A     AACGGAGTGTTTCTAACCGTGAGGTGGCTAAGAGAAGGCGTTGCAAAGCAGAGAAAGGAG
377     AACGGAGTGTTTCTAACCGTGAAGTGGCTAAGAGAAGGCGTTGCAAAGCAGAGAAAGGAG
        ********************** *************************************

12A     AAGGTGGTGTTGATTTAACGGATTGGGTTAGGTTGCGTGTTGCTGAAGGTCGTGGTGCGG
377     AAGGTGGTGTTGACTTAACGGATTGGGTTAGGTTGCGTGTTGCTGAAGGTCGTGGTGCGG
        ************* **********************************************

12A     AATGCTTTGACTCGGTGCTGACACAGGAGATGGGAAGTGATCCTGTTACAGAGAAAGGGA
377     AACGCTTTGACTCGGTGCTGACACAGGAGATGGGAAGTGATCCTGTTACAGAGAAAGGGA
        ** *********************************************************

12A     CGAAAGAAGTTCTTGGGATTGCCTTGAGGTGTATAAGATCTGTGTCTGAGAGA  3173
377     CGAAAGAGGTTCTTGGGATTGCCTTGAGGTGTATAAGATCTGTGTCTGAGAGA  3173
        ******* *********************************************
```

Figure S1a *Bra020876* gene sequence differences between 12A and 377.

(b)

Bra020861

```
12A    -AAAGGGCAGTGCAAGCTTGCATGCCTGCAGGTCGACGATTACGAGATTAGTCATATTCC
377    AAAAGGTCAGTGCAAGCTTGCATGCCTGCAGGTCGACGAT--------------------
        ***** *********************************

12A    CAATAGAAGAGGAAATCTCCACAAGGCTTGAGCATTCACTGAGATCCAATTCCTTGAGAT
377    ------------------------------------------------------------


12A    TGGTCATATTCCCAACGGGGAGCTCCACAAGGCTTGAGCATTCACTGAGATCCAATTTCT
377    ------------------------------------------------------------


12A    TGAGATTGGTCATATTTCCAATAGAAAAAGAGAGCCGTACAACGCTTGAGCATTGGGAGA
377    ------------------------------------------------------------


12A    GATTCAACTTCTCAAGATTAGTCATATTACTAATAGAAGAGGGGACTTCCATAAGGCTTG
377    ----------TACGAGATTAGTCATATTCCCAATAGAAGAGGAAATCTCCACAAGGCTTG
                   * ************** * ***********  *  **** ********

12A    AGCACAGCTTGAGATTCAATTTCTTGAGATTAGTCATATTCCCAATAGAAGAGGAAATCT
377    AGCATCCACTGAGATCCAAATTCTTGAGATTAGTCATATTCCCAA------AGGTGAGCT
       ****     ****** *** *************************      ***  * **

12A    AGGGGGATGTGCTGCAAGGCGATTAAGTTGGGTAACGCCAGGGTTTTCCCAGTCACGACG
377    AGGGGGATGTGCTGCAAGGCGATTAAGTTGGGTAACGCCAGGGTTTTCCCAGTCACGACG
       ************************************************************

12A    TTGTAAAACGACGGCCAGTGCCAAGCTTGCATGCCTGCAGGTCGACGAT-----------
377    TTGTAAAACGACGGCCAGTGCCAAGCTTGCATGCCTGCAGGTCGACGATTATGGTGCCGA
       *************************************************

12A    -----------------------------------------TCTCTAGAGGATCCCCGGG
377    GATGTTCAAGTGCCACAACCATGCCTCCTGCTGGTTACTAATCTCTAGAGGATCCCCGGG
                                                *******************

12A    TACCGAGCTCGAATTCGTAATCATGGTCATAGCTGTTTCCTGTGTGAAATTGTTATCCGC
377    TACCGAGCTCGAATTCGTAATCATGGTCATAGCTGTTTCCTGTGTGAAATTGTTATCCGC
       ************************************************************
```

Figure S1b *Bra020861* gene sequence differences between 12A and 377.
